# INTRAHEPATIC BILIARY STRICTURES WITH UNDERLYING PRE-MALIGNANT BILIARY LESIONS: IS IT TIME TO BUILD GUIDELINES ON DIAGNOSIS AND MANAGEMENT?

**DOI:** 10.1590/0102-672020210002e1613

**Published:** 2021-12-17

**Authors:** Giuliano LA BARBA, Carlo Alberto PACILIO, Cecilia BINDA, Francesca FAPPIANO, Carlo FABBRI, Giorgio ERCOLANI

**Affiliations:** 1 General and Oncologic Surgery Unit, Morgagni-Pierantoni Hospital, AUSL Romagna, Forlì, Italy; 2 Gastroenterology and Digestive Endoscopy Unit, Morgagni-Pierantoni Hospital, AUSL Romagna, Forlì, Italy; 3 Department of Medical and Surgical Sciences, Alma Mater Studiorum, University of Bologna, Bologna, Italy

**Keywords:** Intrahepatic bile ducts, Intrahepatic cholestasis, Carcinoma in situ, Cholangiocarcinoma, Minimally invasive surgical procedures, Ductos biliares intra-hepáticos, Colestase intra-hepática, Carcinoma in situ, Colangiocarcinoma, Procedimentos cirúrgicos minimamente invasivos

## INTRODUCTION AND LITERATURE REVIEW

Biliary intraepithelial neoplasia (BilIN) is defined as a precursor lesion of invasive adenocarcinoma and it can develop both in intrahepatic and extrahepatic bile ducts. BilIN grossly presents as flat lesions but is usually macroscopically and radiologically unidentifiable^10^ ^,^ ^21^. Interestingly, in the last decades due to the spread of imaging techniques, focal intrahepatic biliary strictures (FIHS) are more commonly incidentally founded in clinical practice. It is currently unknown which is their correct management and in what percentage they can harbor pre-neoplastic alterations (as BilIN) or malignancy^20^. Due to the concomitant increase in western countries of intrahepatic cholangiocarcinoma, it would be useful to understand the natural history of FIHS, as they can represent a radiological manifestation of underlying pre-malignant disease: in that perspective, the ideal treatment could be an early surgery to prevent cholangiocarcinoma^3^ ^,^ ^18^. 

Cholangiocarcinoma is the most common biliary tract malignancy, with a well known unfavorable prognosis. Currently, surgery is the only potential curative treatment but, unfortunately, the majority of patients are diagnosed at late stage or are found to be unresectable during explorative laparotomy^3^. Interestingly, during the last three decades, the incidence of the intrahepatic subtype of cholangiocarcinoma has increased in Western Europe^18^.

Cholangiocarcinoma of intrahepatic and extrahepatic bile ducts is known to develop through a multistep carcinogenesis sequence, including biliary intraepithelial neoplasia. BilIN is characterized by a flat or micropapillary growth of atypical biliary epithelium. In 2007 Zen et al^10^. reported the results of the international consensus on its diagnostic criteria and grading. 

In the last 20 years, pathologists have identified two main precursors of invasive cholangiocarcinoma, namely BilIN and intraductal papillary neoplasm of bile duct, both currently included in the WHO classification of tumors of digestive system^10^. As expected, BilIN and intraductal papillary neoplasm have been identified in the bile ducts of patients suffering of primary sclerosing cholangitis, choledochal cyst and hepatolithiasis, all well known risk factors for cholangiocarcinoma. In 2006, Zen et al.^22^ reported a series of 110 patients with biliary neoplasm associated to hepatolithiasis, differentiating cases of BilIN and intraductal papillary neoplasm by cytokeratin and mucin immunoistochemical staining; in that series, cholangiocarcinomas arising from BilIN developed tubular adenocarcinoma, while those arising from intraductal papillary neoplasm developed either tubular or colloid carcinomas; moreover, those latter seemed to have a better survival^16^. Regarding the difference in tumorigenic pathways, BilIN lesions seem to have higher expression of the mucin core protein MUC 1, probably favoring a more invasive phenotype with respect to cholangiocarcinomas harboring form IPNB^1^. Moreover, many studies have supposed BilIN and IPNB to be biliary counterparts of pancreatic intraepithelial neoplasia intraductal papillary neoplasm and intraductal papillary mucinous neoplasm of the pancreas intraductal papillary neoplasm ^8,11,13^. 

Strictly related to the topic of cholangiocarcinoma’s pre-neoplastic lesions, nowadays in clinical practice another issue is emerging, regarding focal undetermined intrahepatic biliary duct strictures.

An appropriate diagnostic evaluation is of paramount importance, because it is currently unknown FIHS’ natural history and if they can harbor malignancy or pre-neoplastic lesions^19^. Based on the accessibility of the biliary tree and the site of the lesion, biliary strictures can be evaluated by endoscopic retrograde cholangiopancreatography or percutaneous transhepatic cholangiography. Both techniques allow sampling of the strictures and therapeutic biliary stent placing. However, biliary brushing cytology, as reported by Trikudanathan et al.^17^ in a recent meta-analysis, has a low sensitivity (not exceeding 43%) in detection of cholangiocarcinoma in primary sclerosing cholangitis patients. The same authors, in another meta-analysis, reported that fluorescence in situ hybridization (FISH) was able to increase the sensitivity up to 68%^12^. 

Another reported technique in the evaluation of indeterminate biliary lesions is the SpyGlass single-operator peroral cholangioscopy^4^ ^,^ ^9^. Woo et al.^19^ reported that overall accuracy of SpyGlass visual assessment and SpyBite biopsy for the diagnosis of malignancy were 96.7% and 73.6%, respectively. Similar results were obtained in a Japanese large prospective multicenter study (95.5% and 70.7%)^9^. Interestingly, this study reported 10 cases of intrahepatic indeterminate biliary lesions, in which accuracy values decreased (80% and 83.3%). A recent meta-analysis by Sun et al.^15^, considering the usefulness of this diagnostic tool in determining malignancy in biliary lesions, reported sensitivity and specificity of visual assessment and visual guided biopsy of 90-87% and 69-98%, respectively. Currently, no study has demonstrated the ability of CT scan or MRCP, as well as endoscopic or intraductal ultrasound, to differentiate benign from malignant intrahepatic strictures.

Regarding treatment, due to the undefined possibility of FIHS to harbor malignancy, it has been advocated that stricture should be resected whenever, during the diagnostic work-up, a malignant diagnosis is suspected or of course ascertained. Yeo et al.^20^, in their review, proposed an interesting algorithm treatment for FIHS: after a two level diagnostic work-up, based on non-invasive and invasive diagnostic tools, in case of supposed benign stricture, cholangioscopy with or without biopsy should be performed. If a benign stricture is confirmed, treatment should be reserved only to symptomatic patients and could be surgical or non-surgical (endoscopic, percutaneous). Regarding the surgical technique, in the last decade, we have assisted to the spreading of laparoscopic liver resection worldwide^1^ and several cases of laparoscopic liver resection for intrahepatic bile duct dilatation with associated hepatolithiasis, a condition quite similar to FIHS, have been described^5^ ^,^ ^6^ ^,^ ^15^. In our centre, we performed in 2019 two cases of left biliary duct stricture who underwent laparoscopic left hepatectomy with final diagnosis of BilIN^10^ ^,^ ^20^. In a retrospective Korean series involving 24 patients who underwent surgical resection for bile duct dilatation without demonstrable mass on pre-operative imaging, Kim et al.^7^ found in half of patients (n=12) malignancy at the final pathology. From a morphological point of view these patients, compared to those with benign bile duct stricture, showed thickening of bile duct wall ≥5 mm and regional lymph node enlargement on CT scan, abrupt cut-off and bile duct separation on cholangiogram. The prospective series by Seo et al.^14^ on percutaneous transhepatic cholangioscopy included 17 patients with FIHS without stones: a histopathologic diagnosis was obtained in all patients (9 adenocarcinomas, 1 squamous cell carcinoma, 2 hepatocellular carcinomas, 2 adenomas and 3 benign strictures). Of the nine with bile duct adenocarcinoma, eight underwent surgery and a curative resection was possible in seven patients. Interestingly, five patients had early-stage bile duct cancer in which cancer invasion was limited to the mucosa or fibromuscular layer and there was no evidence of lymph node metastasis. 

## CONCLUSION

It is raising the fact that FIHS can harbor pre-neoplastic lesions (BilIN). The diagnostic work-up is often inconclusive in order to rule out malignancy, but in literature cholangioscopy with biopsy is emerging as a promising technique to overcome this issue, even if it is not widespread and available in every center as it deserves a specific training and equipment. Whenever the suspicion of malignancy is moderate to high, we believe that surgery is fully justified, as it can represent a kind of preventive treatment of cholangiocarcinoma. Minimally invasive liver resection in this setting could represent the optimal choice. As high level evidence in this topic is still lacking in literature, we call for national and international registers to address the issue of diagnosis and management of focal intrahepatic undetermined strictures. 
